# A combined spectroscopic and molecular modeling Study on structure-function-dynamics under chemical modification: Alpha-chymotrypsin with formalin preservative

**DOI:** 10.3389/fchem.2022.978668

**Published:** 2022-08-31

**Authors:** Pritam Biswas, Aniruddha Adhikari, Uttam Pal, Susmita Mondal, Dipanjan Mukherjee, Ria Ghosh, Rami J. Obaid, Ziad Moussa, Sudeshna Shyam Choudhury, Saleh A. Ahmed, Ranjan Das, Samir Kumar Pal

**Affiliations:** ^1^ Department of Microbiology, St. Xavier’s College, Kolkata, India; ^2^ Department of Chemical, Biological and Macro molecular Sciences, S. N. Bose National Centre for Basic Sciences, Kolkata, India; ^3^ Technical Research Centre, S. N. Bose National Centre for Basic Sciences, Kolkata, India; ^4^ Department of Chemistry, Faculty of Applied Sciences, Umm Al-Qura University, Makkah, Saudi Arabia; ^5^ Department of Chemistry, College of Science, United Arab Emirates University, Al Ain, Abu Dhabi, United Arab Emirates; ^6^ Chemistry Department, Faculty of Science, Assiut University, Assiut, Egypt; ^7^ Department of Chemistry, West Bengal State University, Barasat, Kolkata, India

**Keywords:** amino acid modification, chymotrypsin, formalin, cross-linking, spectroscopy, molecular docking

## Abstract

Enzyme function can be altered *via* modification of its amino acid residues, side chains and large-scale domain modifications. Herein, we have addressed the role of residue modification in catalytic activity and molecular recognition of an enzyme alpha-chymotrypsin (CHT) in presence of a covalent cross-linker formalin. Enzyme assay reveals reduced catalytic activity upon increased formalin concentration. Polarization gated anisotropy studies of a fluorophore 8-Anilino-1-naphthalenesulfonic acid (ANS) in CHT show a dip rise pattern in presence of formalin which is consistent with the generation of multiple ANS binding sites in the enzyme owing to modifications of its local amino acid residues. Molecular docking study on amino acid residue modifications in CHT also indicate towards the formation of multiple ANS binding site. The docking model also predicted no change in binding behavior for the substrate Ala-Ala-Phe-7-amido-4-methylcoumarin (AMC) at the active site upon formalin induced amino acid cross-linking.

## Introduction

Covalent modifications introduce addition or removal of groups from the amino acid residues of a protein altering its structure and function. Formalin, simplest aldehyde is flammable and highly reactive and polymerizes readily under normal temperature and pressure. Formalin is known to react with a plethora of functional groups in small peptides and other substrates to generate covalently modified products (Fraenkel-Conrat and Olcott, 1948; Hoffman et al., 2015) which can include both intramolecular and intermolecular cross-linked species. Formalin due to its high reactivity and permeability into cells and tissues has led to its use in numerous applications in medicine and medical biotechnology. (Hoffman et al., 2015). From a chemical standpoint, formalin can react with biological nucleophiles such as protein and DNA and facilitates *in vitro* formation of intra-strand and DNA-protein cross links. (Brutlag et al., 1969; Metz et al., 2004; Hoffman et al., 2015). Formalin related reactions in cells are likely responsible for its toxic/carcinogenic effects, however the precise chemistry of such reactions and their cellular prevalence is still not clearly defined. (Lu et al., 2010). Studies have indicated that formalin causes crosslinking of proteins, involving the formation of a methylene bridge between two proximal amino acids. (Feldman, 1973; Metz et al., 2006). Formalin is widely used as a fixative, as it chemically modifies proteins irreversibly, although the first stages of such reactions are reversible, such as the reaction of an aldehyde with a free thiol to form a thioacetal, RS-C(OH)H_2_, or with an amine to form a Schiff-base adduct > C=N-. (Wait et al., 2011). Pioneering works by McGhee and Von Hippel (McGhee and Von Hippel, 1977) and a recent study by Kamps *et al.* (Kamps et al., 2019) as well Tayri-Wilk et al. (2020) on the reactions of nucleosides/nucleotides and amino acids with formalin have envisaged to understand the chemistry of such reactions.

To have a better understanding how formalin influence health and diseases, it is important to investigate the correlation between formalin induced structural modification and function of biological macromolecules. Previous studies on interaction of formalin with biological macromolecules were mostly restricted to residue modification studies. (Metz et al., 2004; Metz et al., 2006; Lu et al., 2008; Metz et al., 2020). These studies, however, revealed very little information regarding residue modification and its correlation to protein function and molecular recognition. In the present study, residue modification of a common digestive enzyme *a*-chymotrypsin (CHT; EC 3.4.21.1) by formalin was correlated with its catalytic activity and molecular recognition of a fluorescence probe (ANS) using steady state and pico-second resolved fluorescence spectroscopy, circular dichroism (CD), Dynamic light scattering (DLS). Here, we also utilize structural bioinformatics and develop a molecular docking model for understanding the effect of residue modification on molecular recognition.

## Materials and methods

### Materials

α-Chymotrypsin (CHT) (*Bostaurus*) (≥40 U/mg), Ala-Ala-Phe-7-amido-4-methylcoumarin (AMC) (≥98%), 8-Anilino-1-naphthalenesulfonic acid (ANS) (≥97%) were purchased from Sigma (Saint Louis, United States). Formaldehyde (37%) was purchased from Merck. *a*-Chymotrypsin, AMC and ANS solutions were prepared in phosphate buffer (10mM, pH 7.0) using water from Millipore system.

### Sample preparation

Formalin modified CHT was prepared by incubating the enzyme at different formalin (1%, 2%, 4%) concentrations at 4°C for 5 h, which was dialysed before experimentation.

ANS is a well-known protein probe, and is a drug mimic. The ANS-CHT complex was prepared by mixing ANS (0.5 µM) with CHT (5 µM) in phosphate buffer (pH 7.0). The mix was stirred continuously for 5 h at 4°C, which was filtered extensively to remove the free probe. (Pal et al., 2002).

### Circular dichroism spectroscopy

Circular dichroism (CD) spectroscopy analysis of CHT was conducted with 2 µM CHT solution incubated with different concentrations of formalin (1%, 2%, 4%) using a quartz cuvette of 1 mm path length in a JASCO 815 spectrometer. The deconvolution of the CD signals into relevant secondary structure was done by CDNN software. (Böhm et al., 1992).

### Dynamic light scattering

Dynamic light scattering (DLS) measurements was done with 1.5 µM CHT solution incubated with different concentrations of formalin using a Nano S Malvern instrument employing a 4 mW He-Ne laser (*λ*_ex_ = 632 nm).

### Enzyme activity assay

Enzymatic activity of CHT was conducted with 0.5 µM of CHT at different formalin concentrations (1%, 2%, 4%). The substrate Ala-Ala-Phe-AMC (*λ*_max_ = 325 nm) was cleaved to produce 7-amido-4-methyl-coumarin (*λ*_max_ = 370 nm), the absorbance of which was monitored in Shimadzu Model UV-2600 spectrophotometer.

### Time correlated single photon counting measurement

Picosecond-resolved fluorescence spectroscopic study were carried out using a commercial time correlated single photon counting (TCSPC) from Edinburgh instruments. The picosecond excitation pulses from the picoquant diode laser were used at 375 nm for the excitation of ANS with an instrument response function (IRF) of 80 ps at different formalin concentration. Fluorescence photons were detected by a microchannel-plate-photomultiplier tube (MCP-PMT; Hamamatsu Photonics, Kyoto, Japan). The time-resolved instrument provided a time resolution of 20 ps, which is one-fourth of the instrument response (IRF) for the detection of time constants after de-convolution of the IRF. For all decays, the emission polarizer was set at 55° (magic angle) with respect to the polarization axis of the emission beam. The observed fluorescence transients were fitted using a nonlinear least-squares fitting procedure. The details of which can be found elsewhere. (Sardar et al., 2015; Bagchi et al., 2016).

### Polarization-gated anisotropy measurements

For the anisotropy measurements (r(*t*)), the emission polarizer was adjusted to be parallel and perpendicular to that of the excitation. Time-resolved anisotropy, r(*t*), was determined from the following equation:

$$r\left(t\right)=\frac{I_{VV}\left(t\right)-G\;.\;I_{VH}\left(t\right)}{I_{VV}+2\;.\;G.I_{VH}\left(t\right)}$$ (1)

where *I*_VV_(*t*) and *I*_VH_(*t*) are parallel and perpendicular polarized fluorescence decays of the dye, respectively, recorded using a vertically polarized excitation light. *G* is an instrument grating factor, which is an instrument and wavelength dependent correction factor to compensate for the polarization bias of the detection system and its magnitude was obtained by a long tail matching technique. (Schröder et al., 2005; O'Connor, 2012)

### Solvent accessible surface area

Solvent accessible surface area (SASA) is a measure of formation of contacts between the atoms on the surface of the protein and the solvent molecules. Thus, solvent accessibility provides a better insight on the amino acid residues of the enzyme (CHT) vulnerable to formalin induced modification. The validation and selection of the appropriate protein data bank (PDB) structure of CHT was made according to Chakraborty *et al.* (Chakraborty et al., 2020). Solvent accessible surface area (SASA) of CHT (PDB ID: 1CGJ) is computed using the PyMOL software (Educational version). The catalytic and residues of ANS binding site were identified as the prime targets for the SASA analysis.

### Residue modification and molecular docking

Solvent exposed residues susceptible to formalin modification were modified followed by minimization of the modified residues using Schrodinger Maestro 2018-1 (Academic release). All covalent modifications were conducted according to Kamps et al. (2019) Targeted molecular docking studies of the modified amino acid residues were performed using AutoDock Vina (Trott and Olson, 2010) to study the influence of formalin induced covalent modification on substrate and molecular recognition of ANS by CHT. Protein and ligand preparation were done using AutoDock Tools 1.5.6. Polar hydrogens were added, the bond order and the subsequent renumbering of the newly added polar hydrogen were done. To add partial charge to the CHT molecule Gasteiger charges were added accordingly. The docking grid was localized on the substrate and ANS binding site. Furthermore, SASA analyses of the bound ANS were also performed according to the methodology described in the previous section.

## Result & discussion

### Structural analysis

**FIGURE 1:**
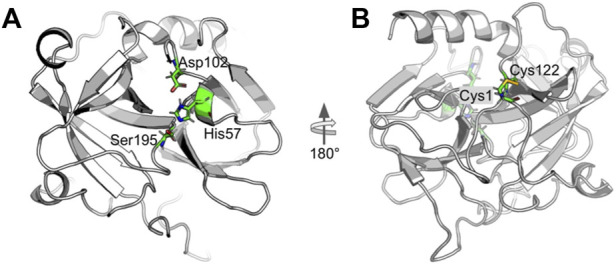
Cartoon representation of alpha-Chymotrypsin (CHT) [PDB ID:1CGJ]. **(A)** The coloured stick representation of the active site catalytic triad; showing the active site residues [His-57; Asp-102; Ser-195]. **(B)** At the N-terminal, the coloured stick representation [Cys-1; Cys-122] shows the molecular recognition site [External ANS binding site]. This site is located almost opposite to the catalytic site.

α-Chymotrypsin (CHT) is a proteolytic enzyme of the class serine protease (EC 3.4.21.1) which is associated with hydrolysis of peptide bonds in the mammalian digestive system. The catalytic triad of CHT is located in the hydrophobic S1 pocket of CHT, (Ma et al., 2005; Andersson et al., 2017). Figure 1 shows the 3D structure of CHT. Figure 1A highlights the catalytic triad comprising the residues histidine-57; aspartate-102 and serine-195. The active site residue serine-195 serves as probe binding site (Banerjee and Pal, 2008). While, the site at cys-1-122 disulfide bond (Figure 1B) also serves as one of the probe (ANS) binding sites and is located almost opposite to the catalytic site (Johnson et al., 1979).

### Enzymatic activity assay

**FIGURE 2:**
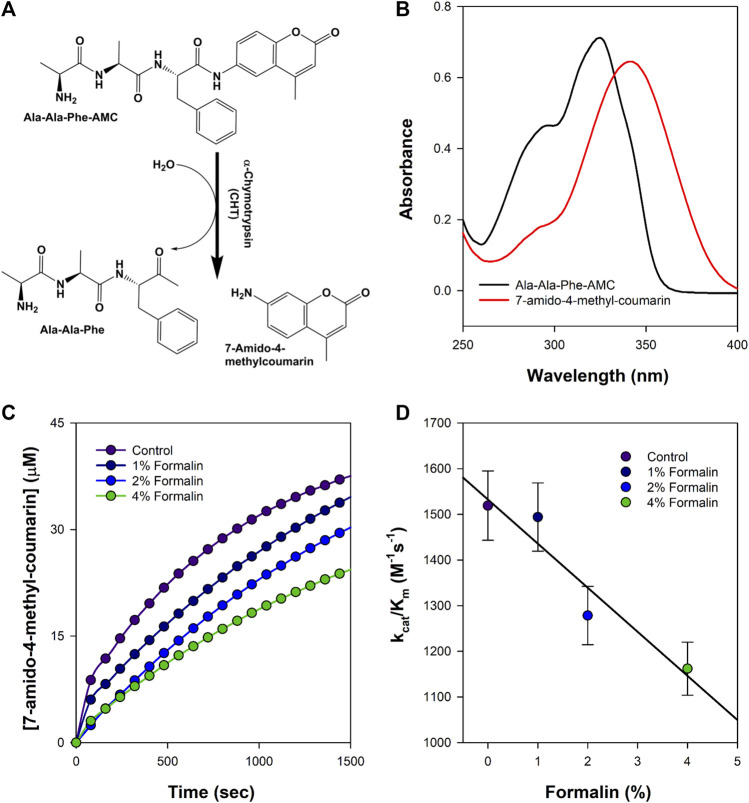
Schematic representation of the formalin treated enzyme catalysis. **(A)** Simple reaction outline catalysed by CHT; the substrate AMC hydrolysed to produce 7-amido-4-methyl-coumarin. **(B)** Absorbance spectra of the substrate AMC and product 7-amido-4-methyl-coumarin during catalysis. **(C)** Product formation at different representative formalin concentration; diminishing product formation with increased formalin concentration. **(D)** Apparent catalytic efficiency (k_cat_/K_m_) at different representative formalin concentration; retarding catalytic efficiency is consistent with decreasing product formation with increasing formalin concentration (Experiments were performed in triplicate).

The catalytic activity of CHT was measured with different AMC (substrate) concentrations (5–400 µM) at varying formalin concentrations (1%–4%). To ensure the reaction between the enzyme and formalin, CHT was kept for 5 h at 4°C at the different ascribed concentrations of formalin, followed by dialysis of the enzyme before experimentation. Formalin is a chemical modification agent which causes irreversible covalent inhibition of CHT. CHT belongs to the class of serine protease that catalyzes hydrolysis of AMC to 7-amido-4-methy-coumarin (Figures 2A,B). Figures 2C,D illustrate the effects of increasing formalin concentration on the product formation and the apparent catalytic efficiency. A reduction in the apparent catalytic efficiency was observed (Table 1) with increasing formalin concentration reflecting on reduced catalytic activity which likely originates from a possible modification of the substrate binding site. A reduction in the apparent catalytic efficiency (k_cat_/K_m_) with increasing formalin concentration indicates a reduced catalytic activity which likely originates from a possible modification of the substrate binding site as indicated from previous reports. (Marini and Martin, 1971; Martin et al., 1972). however, it did not escape our attention that the reduced catalytic activity could also be caused due to enzyme aggregation and precipitation due to formalin modification. Hence, a structural analysis was conducted.

**TABLE 1:** Kinetics of CHT catalyzed hydrolysis of Ala-Ala-Phe-AMC at different formalin concentration.

Formalin concentration (%)	K_m_ (M)	V_max_ (μM/s)	K_cat_ (s^−1^)	k_cat_/K_m_ (M^−1^s^−1^)
Control	(7.9 ± 0.39)×10^−5^	0.060 ± 0.003	0.12 ± 0.006	1,518.98 ± 75.94
1	(8.3 ± 0.41)×10^−5^	0.062 ± 0.003	0.12 ± 0.006	1,493.97 ± 74.69
2	(9.7 ± 0.48)×10^−5^	0.060 ± 0.003	0.12 ± 0.006	1,278.35 ± 63.91
4	(1.05 ± 0.05)×10^−4^	0.061 ± 0.003	0.12 ± 0.006	1,161.90 ± 58.09

### Secondary and globular tertiary structure analysis

**FIGURE 3:**
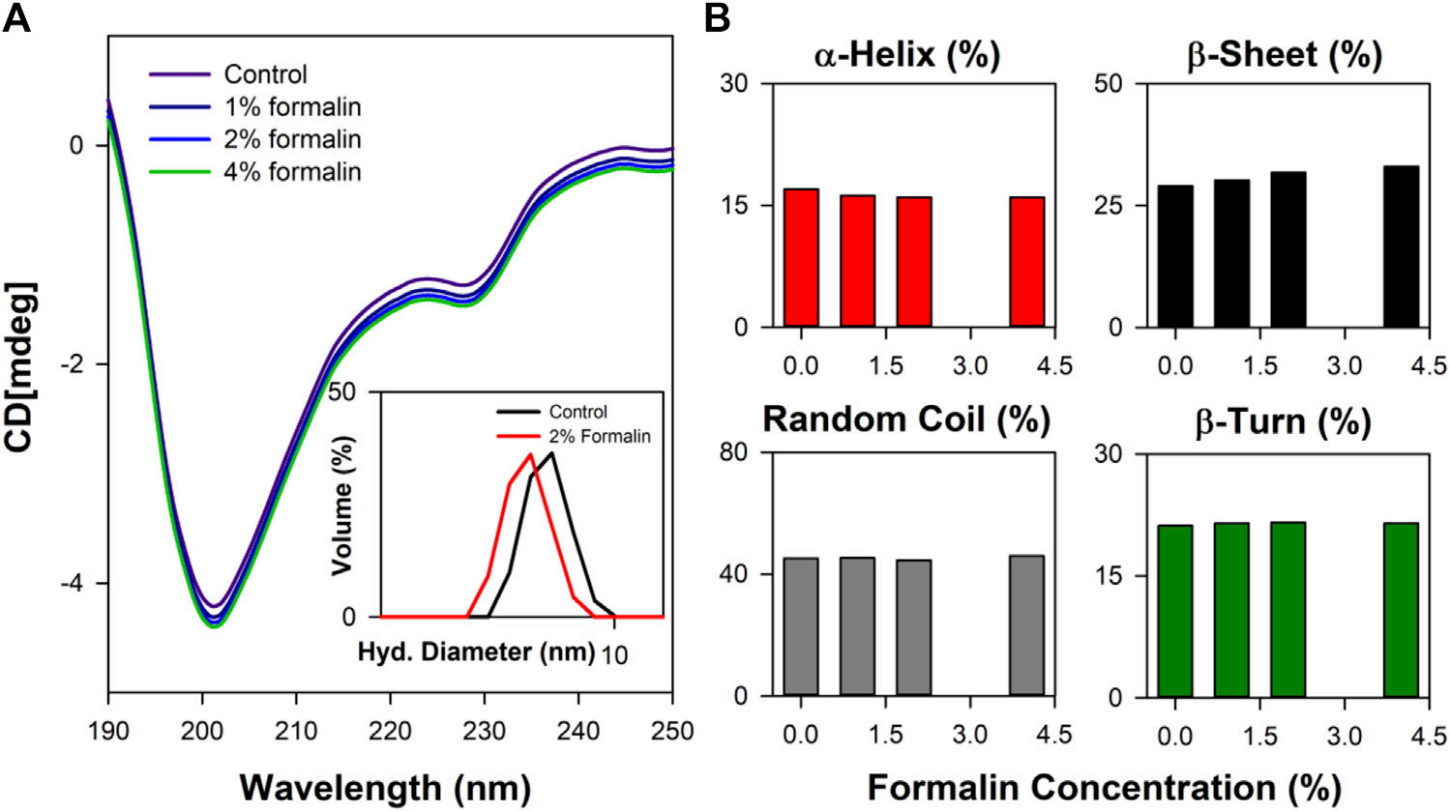
Far UV CD spectroscopy study of CHT. **(A)** The far UV CD spectra representing structural integrity of formalin treated CHT. The inset shows the DLS spectra of CHT at representative formalin concentration. **(B)** Percentage composition of secondary structure shown as bar plots. Analysis of secondary structure indicated structural stability within the experimental setup.

Close analysis of the 3D structure of CHT (Figure 1) shows that the enzyme consists of anti-parallel *ß*-sheets which are highly distorted, forming very short irregular strands. Such conformation can lead to a shift in the negative band from the ideal *ß*-sheet position (210–220 nm) towards 200 nm. The effect of formalin induced modification on CHT was quantified by circular dichroism (CD) spectroscopy in the far UV region. The far UV spectra of CHT were characterized by a minimum at 202 nm with no positive band (Figure 3). The spectral data for all formalin concentration (1%, 2%, and 4%) was recorded. Since, 4% formalin corresponds to 1.3 M, the reliability of the CD spectra can be questioned. So, we recorded the CD voltage for all the corresponding CD spectra and found the CD voltage to be < 500 V, which was well within the acceptable range (<700 V) and hence, the CD spectral data was considered reliable. Upon de-convolution, a 4% increase in anti-parallel *ß*-sheet content was observed along with a nominal decrease in *α*-helix and insignificant changes in parallel *ß*-sheet, turns and random coils (Table 2). These results indicate an insignificant change in the secondary structure of the enzyme reflecting on its structural stability. The effect of formalin on the globular tertiary structure of CHT was monitored by dynamic light scattering (DLS). The DLS study (Figure 3A Inset) reveals the hydrodynamic diameter of CHT to be ∼7 nm, which agrees with previous studies (Shaw et al., 2007; Banerjee and Pal, 2008). Upon treatment with 4% formalin, a decrease in the hydrodynamic diameter of CHT from ∼7 nm (control) to ∼5 nm was noted (Supplementary Table S1) indicating no CHT aggregation due formalin treatment within the experimental conditions.

**TABLE 2:** Secondary structure analysis of alpha-Chymotrypsin in presence of formalin.

Formalin conc (%)	% *α*-helix	% *ß*-sheet	% Random coil	% *ß*-turn
Control	17.00	29.00	45.20	21.20
1	16.20	30.20	45.30	21.50
2	16.00	31.80	44.60	21.60
4	16.00	33.00	46.00	21.50

### Picosecond-resolved fluorescence studies

**FIGURE 4:**
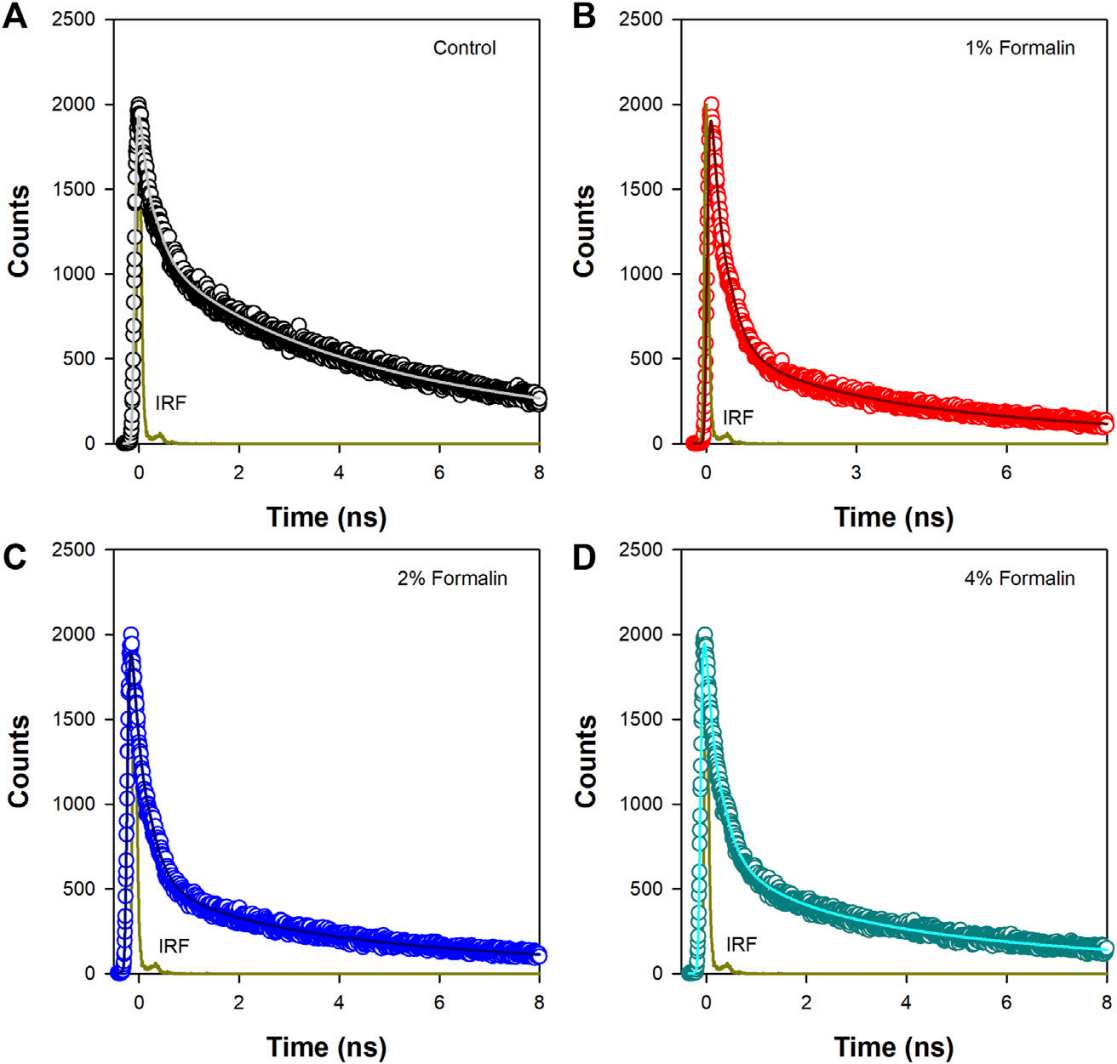
Time resolved fluorescence transients of ANS in **(A)** Pure CHT (control) and at varying formalin concentrations **(B–D)**.

In water fluorescence of ANS decays rapidly with a time constant of 0.25 ns. (Collini et al., 2003). The fluorescence transients of ANS in CHT at different formalin concentrations (Figure 4) are characterized by three-time constants (Table 3) indicating its binding to the enzyme. The faster component $\left(\tau_{1}\right)$ of few hundreds of picoseconds (∼0.25 ns) originates due to free ANS in water. (Collini et al., 2003). ANS bound to CHT reveals two different lifetimes (∼2.1 and ∼7.1 ns) which may be ascribed to two different binding sites in the enzyme; the shorter lifetime $\left(\tau_{2}\right)$ corresponds to an external binding site partially exposed to water, whereas, the longer one $\left(\tau_{3}\right)$ may be ascribed to an internal binding site located inside the enzyme which is screened from water. Upon an increase in formalin content, the longer time constant remains unchanged whereas the shorter fluorescence lifetime $\left(\tau_{2}\right)$ decreases to 1.4 ns (Table 3). This may be ascribed to structural modification in the external binding site leading to increased exposure of the probe (ANS) to water resulting in a shortening of the shorter fluorescence lifetime $\left(\tau_{2}\right)$ . This observation for CHT in ANS is not new one. Zewail et al. (2002) has reported at pH 3.6 in CHT, there is 39% amplitude weighted average of ANS long time which reduces to 30% at pH 6.7. X-ray crystallographic structure reveals at higher pH structural perturbation favours the exposure of the ANS binding site of CHT towards bulk hydration, compared to that of lower pH. This further supports our observation.

**TABLE 3:** Fluorescence lifetimes of ANS in *a*-Chymotrypsin in presence of formalin.

Formalin conc	τ_1_ (ps)	τ_2_ (ps)	τ_3_ (ps)	τ_avg_ (ps)
0	0.235 (55)	2.11 (14)	7.05 (31)	2.71
1%	0.259 (78)	1.94 (10)	7.21 (12)	1.29
2%	0.254 (77)	1.76 (10)	7.19 (13)	1.37
4%	0.240 (72)	1.41 (13)	7.22 (15)	1.49

### Polarization-gated fluorescence anisotropy

**FIGURE 5:**
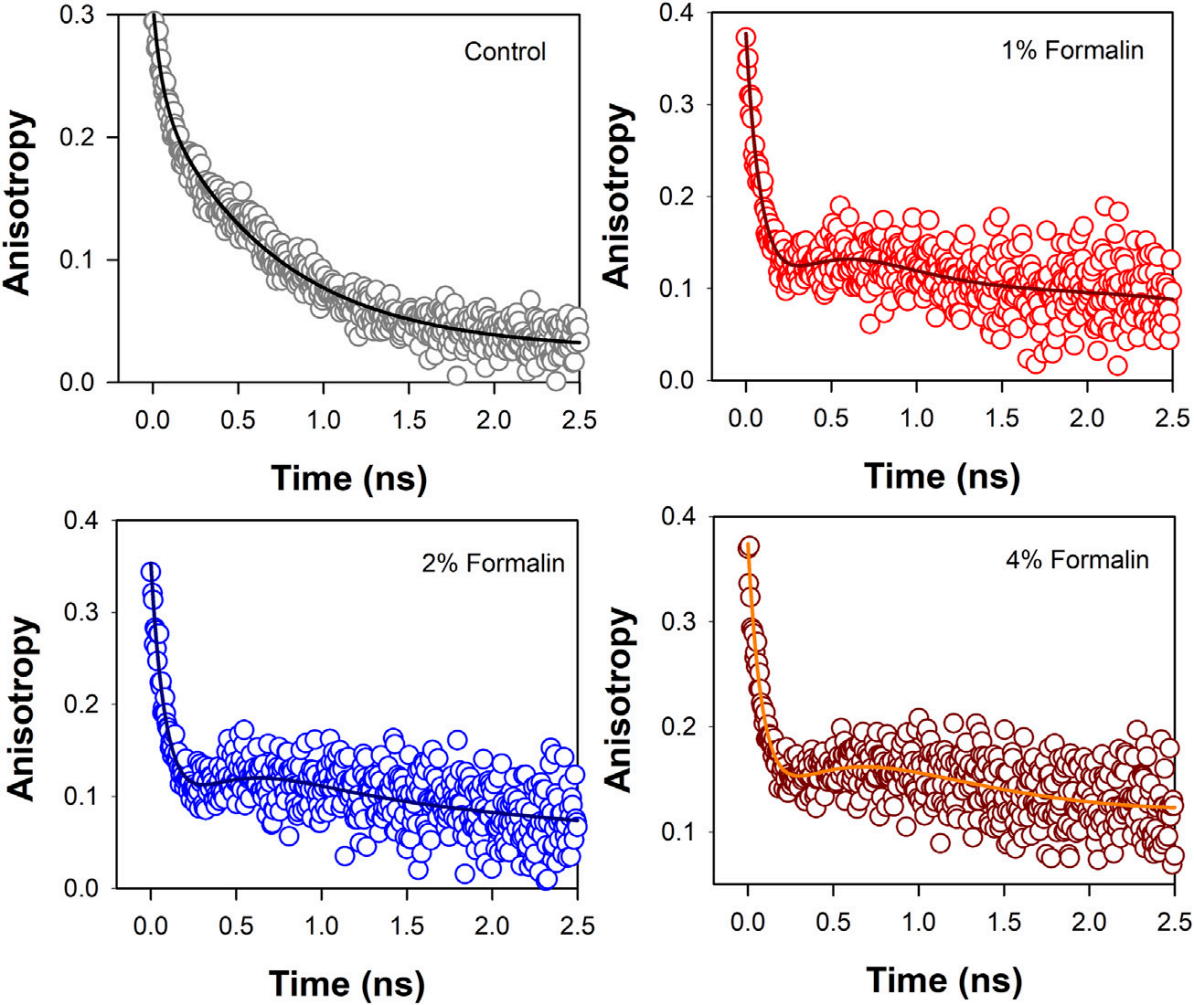
Anisotropy decay [r(t)] of ANS in CHT in absence and presence of formalin.

Polarization-gated fluorescence anisotropy of ANS in CHT was measured (Figure 5) at different formalin concentrations to monitor changes around the ANS binding sites of the enzyme. The anisotropy decay in CHT (Figure 5; control) is characterized by three rotational time constants of ∼56 ps (θ_1_), ∼712 ps (θ_2_) and 30 ns (θ_3_), respectively, (Table 4). The faster (θ_1_) and the relatively slower correlation time (θ_2_) is attributed to orientational motion of the dye in water (Pal et al., 2002) and that (ANS) bound to an external binding site in CHT (Cys-1-122) (Johnson et al., 1979), respectively. On the other hand, the much longer time constant (θ_3_) originates from rotational motion of the ANS/CHT complex where the probe is strongly bound to an internal binding site of the enzyme so that it rotates with the enzyme as a protein/dye complex. (Johnson et al., 1979). Upon interaction with formalin the fluorescence anisotropy decay of ANS in CHT is remarkably modified and a dip-rise pattern becomes evident (Figure 5) owing to heterogeneous distribution of ANS in different microenvironments with different fluorescence lifetimes and associated rotational correlation times. (Sinha et al., 2008; Paul and Guchhait, 2010; Lakowicz, 2013). When a fluorophore exists in different microenvironments, its anisotropy decay, r(t) can be expressed as: (Broos et al., 1995; Paul et al., 2015):

$$\text{r}\left(\text{t}\right)=\sum_{\text{m}}{\text{f}}_{\text{m}}\left(\text{t}\right){\text{r}}_{\text{m}}\left(\text{t}\right)\;$$ (2)

where the fractional intensity of the *m*th component at any time t is given by

$$f_{m}\left(t\right)=\frac{\alpha_{m}\exp\left(-\frac{t}{\tau_{m}}\right)}{\sum_{m}\alpha_{m}\exp\left(-\frac{t}{\tau_{m}}\right)}$$ (3)

α_m_ is the amplitude corresponding to the fluorescence lifetime $\tau_{m}$ at t=0 and

$$r_{m}\left(t\right)=r_{0m}\exp\left(-\frac{t}{\theta_{m}}\right)$$ (4)

where θ_m_ is the single rotational correlation time of the *m*th component and r_0m_ is corresponding initial anisotropy.

**TABLE 4:** Time-resolved Fluorescence anisotropy parameters.

System	θ1/ns	θ2/ns	θ3/ns
ANS in CHT*	0.056 (43)	0.712 (45)	30 (12)
ANS in CHT+1% formalin	0.098 (52)	0.584 (39)	30 (9)
ANS in CHT+2% formalin	0.094 (56)	0.425 (38)	30 (6)
ANS in CHT+4% formalin	0.092 (62)	0.244 (36)	30 (2)

* The anisotropy decay of ANS, in CHT, is fitted by the following equation.
$$r\left(t\right)=r_{0}\left(\beta_{1}\exp\left(-\frac{t}{\theta_{1}}\right)+\beta_{2}\exp\left(-\frac{t}{\theta_{2}}\right)+\beta_{3}\exp\left(-\frac{t}{\theta_{3}}\right)\right)$$

Where r_0_ is initial anisotropy, β_i_s and θ_i_s are the amplitudes and corresponding rotational time constants, respectively. The longest rotational correlation time is kept constant at 30 ns. In presence of formalin, the anisotropy decays are fitted with Eq. 2 as described in the text.

The analysis of the dip and rise anisotropy decay (Figure 5) using equation (2) shows presence of three rotational correlation times (Table 4) where the fastest (θ_1_) and the longest (θ_3_) rotational correlation times remain almost unchanged with a variation in the formalin content. The longer rotational relaxation constant is not an arbitrary one. In order to investigate the longer time constants which corresponds to the global tumbling motion of the entire CHT-ANS complex, we have employed modified Stokes-Einstein-Debye equation where τ_r_ is defined as:

$$\tau_{r}=\frac{\eta_{m}v_{m}fc}{k_{B}T}$$ (5)

Here, $\tau_{r}$ is the rotational time constant of the probe in the enzyme; $k_{B}$ and $v_{m}$ are the Boltzmann constant and molecular volume of the probe at an absolute temperature T; c represents solute−solvent coupling constant, whereas f is the is the shape factor. Here we have considered the values of *C* and *f* to be unity. $v_{m}$ is calculated from the hydrodynamic diameter which lies ∼3.2 nm $k_{B}$ is the Boltzmann constant and considered to be 1.38*10^−23^ J K^−1^ $\eta_{m}$ is considered as 10^−3^Pa. S. By substituting all the values, the obtained rotational time constant ∼30 ns.

However, a gradual decrease in the intermediate correlation time (θ_2_) from 712 to 244 ps with increasing formalin content is observed which may be attributed to covalent modification of the amino acid residues in the enzyme by formalin. As a result of such residue modification the external binding site of ANS in the enzyme likely becomes more exposed to water giving rise to faster rotational relaxation of the dye (ANS).

### Solvent accessible surface area

**FIGURE 6:**
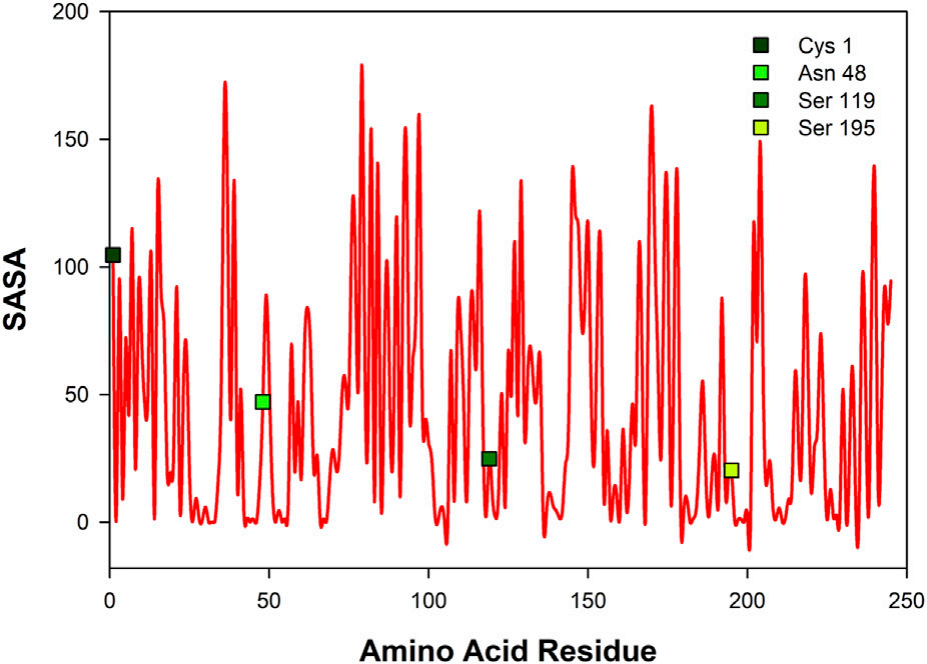
Solvent accessible surface area (SASA) of CHT [PDB ID: 1CGJ]. Four different residues corresponding to the catalytic site and molecular recognition were selected [Cys-1; Asn-48; Ser-119; Ser-195] for formalin induced modification.

Figure 6 shows solvent accessible surface area of CHT (1CGJ). It becomes evident that several amino acid residues of CHT are exposed to solvent and hence, are vulnerable to formalin induced modification. However, we have restricted our study to the substrate and the probe (ANS) binding sites in the enzyme. At the catalytic S1 pocket of CHT which also serves as the buried internal site of ANS binding, Ser-195 is solvent exposed and contains a free hydroxyl group making it susceptible to formalin induced modification. (Kamps et al., 2019). Furthermore, study of the external ANS binding, which is at Cys-1-122 (Johnson et al., 1979) site indicates Cys-1 to be vulnerable to modification by formalin owing to its high extent of solvent exposure and the presence of a free amino (-NH_2_) group. In contrary, Cys-122 has no free amino (NH_2_) group making it almost immune to formalin induced modification. This study also reveals the presence of susceptible residues Asn-48 and Ser-119 within 3 Å of the ANS binding site, making them suitable candidates for our study.

### Residue modification and molecular docking analysis

**FIGURE 7:**
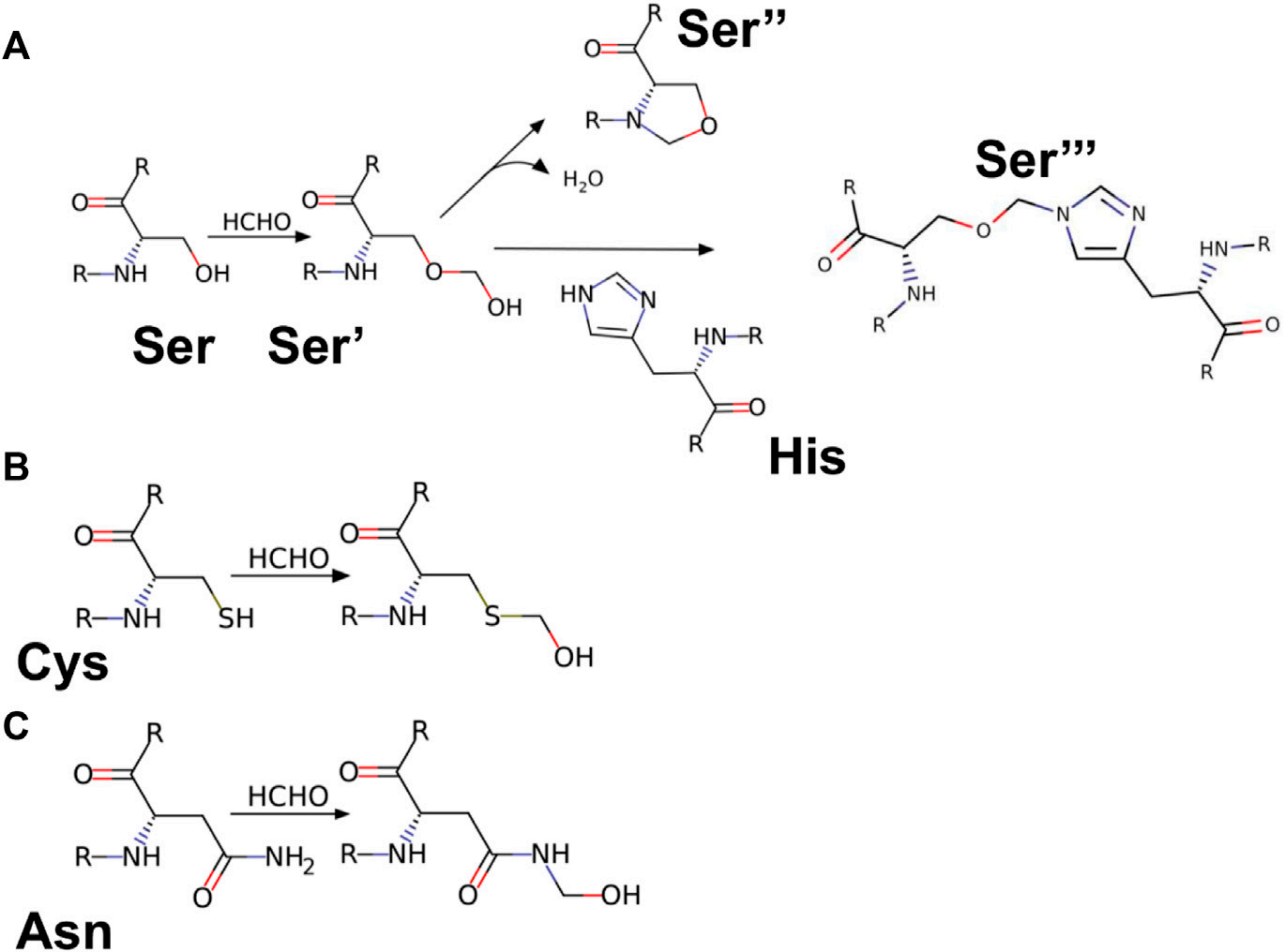
Reaction products of HCHO with different amino acids. **(A)** Amino acid Serine (Ser) reacting with HCHO form methylol derivative [Ser’]; which undergo cyclisation to form oxazolidine adduct [Ser’‘]; the methylol derivative can react with nearby amino acid [case in point amino acid Histidine (His)] to form a crosslink derivative [Ser’’‘]. **(B,C)** Reaction of HCHO with amino acids Cysteine (Cys) and Asparagine (Asn) forming their respective methylol derivative.

To study the influence of residue modification on the binding of substrate (AMC) and its ability to recognize molecular binding partner (ANS) a molecular docking model was designed, the residues selected from the SASA study (Ser-195; Cys-1; Asn-48; Ser-119) were subjected to residue modification according to Kamps et al. (2019) Figure 7 shows various possible modifications due to reaction with formalin. From Figure 7A it becomes evident that serine has three possible modified outcomes. The free hydroxyl (OH) group undergoes modification forming a methylol adduct (Ser’), which can undergo internal cyclization, forming oxazolidone adduct (Ser’‘) or forming a crosslink with the nearby His-57 residue (Ser’’’). Similarly, Cys-1 and Asn-48, undergo modifications forming a methylol adduct at the free thiol (-SH) or amino (-NH_2_) group (Figures 7B,C). However, due to the formation of a disulfide bond between Cys-1 and Cys-122, no thiol group is available for formalin induced modification. Cys-1 being the N-terminal amino acid, contains a free amino group (-NH_2_) which reacts with the formalin.

**FIGURE 8:**
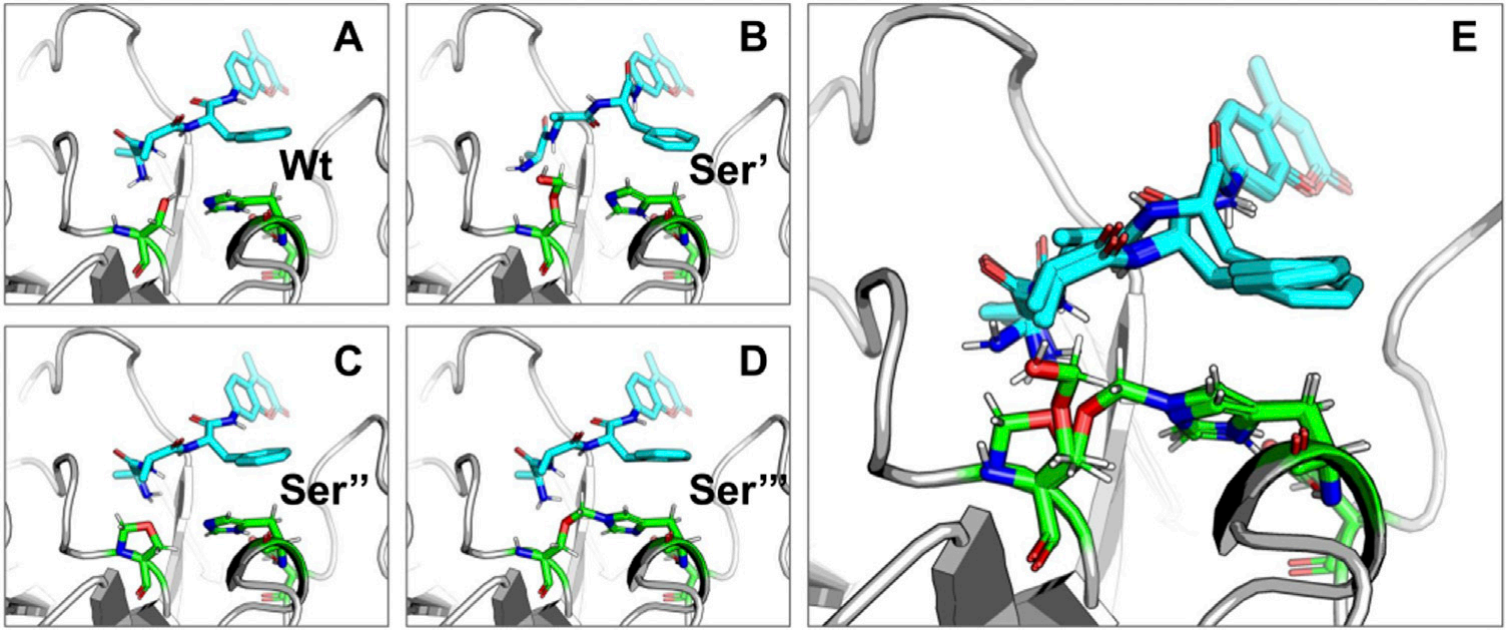
Binding of substrate (AMC) with Wt and HCHO modified CHT as obtained by molecular docking simulations. **(A–D)** Different modes of AMC binding with CHT on modification of the active site Serine residue (Ser-195) as mentioned previously [Wt indicates the wild type Ser-195 residue]. **(E)** Overlapping view of the different binding interactions; residue modification causes no major change in binding mode of AMC with CHT.

**FIGURE 9:**
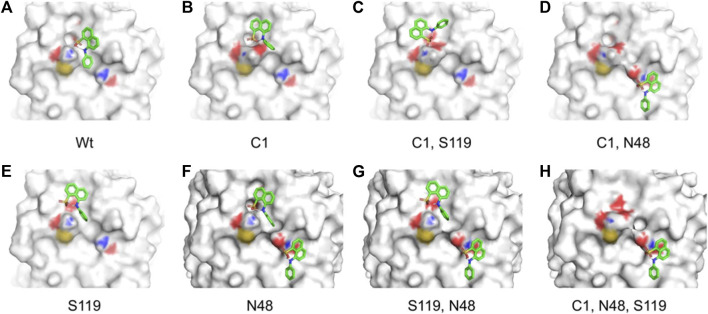
Different binding modes of ANS with CHT (external site) as obtained by molecular docking simulation. **(A–H)** Best binding modes of ANS on residue modification [Cys-1; Asn-48; Ser-119] as mentioned previously; extensive change in binding modes resulting in heterogeneity of ANS binding is evident.

**FIGURE 10:**
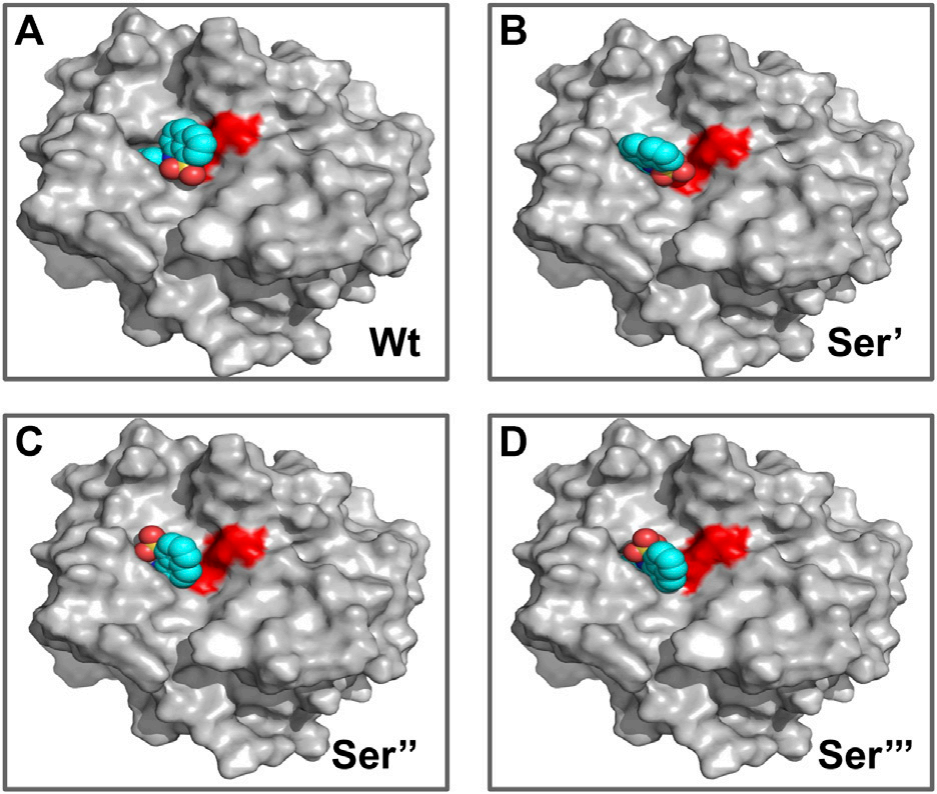
Different binding modes of ANS with CHT (internal site) as obtained by molecular docking simulation. **(A–D)** ANS binding modes with CHT on active site serine (Ser-195) modification as previously mentioned. [Wt indicates the wild type/unmodified CHT].

Targeted molecular docking was performed on the modified CHT structure. It should be noted that only the lowest energy binding poses were selected for the study. Figures 8–10 illustrate different ligand bound structures. It becomes evident from the docking studies that residue modification at the catalytic site has no influence on binding of CHT to the substrate (AMC) (Figure 8). Furthermore, a study of the van der Waals surface indicated no major structural distortions of the binding pocket (Supplementary Figure S3). The possible formation of Ser-195-His-57 crosslink upon formalin treatment may cause a disruption of the serine protease activity at the catalytic triad; thus, impairing the product formation and release, leading to reduced catalytic activity.

Our time resolved studies indicate the presence of two probe binding sites-internal binding site (buried), external binding site (exposed); which was described in previous studies (Johnson et al., 1979; Banerjee and Pal, 2008). Targeted molecular docking of the probe ANS and CHT was performed at the two binding sites, namely, the buried internal site at the S1 pocket and an external binding site at Cys-1-122 di-sulfide bond (Johnson et al., 1979; Banerjee and Pal, 2008). It becomes evident from Figure 9 that, formalin induced residue modification at the external binding site of ANS may lead to the generation of multiple probe (ANS) binding sites and a heterogeneous probe binding distribution which is indeed reflected by the dip-rise pattern in the anisotropy decay (Figure 5). Docking simulations of CHT modified at the external binding site Cys-1 and its nearby susceptible residues Ser-119, Asn-48 generated binding models (Figures 9B–H) which deviate from the unmodified CHT (Figure 9A). Although molecular docking does not provide pronounced solvent effects in protein/ligand interactions, however, by probing the solvent accessibility of the bound ligand (ANS), solvent effects in protein-ligand interactions can be elucidated from molecular docking studies. SASA analysis of the bound probe found a diverse range of solvent effects upon formalin modification of the external site. The varied solvent effects ranged from mimicking the unmodified ANS binding site [modification at Cys-1; 1,244 Å^2^ and Ser-119; 1,236 Å^2^] and lesser degree of solvent accessibility [modifications at Asn-48; 1150Å^2^] to experiencing higher solvent effects [modifications in conjunction at Cys-1 Asn-48; (1374Å^2^), Cys-1 Ser-119; (1402Å^2^), Ser-119 Asn-48; (1369Å^2^), Cys-1 Asn-48 Ser-119; (1360Å^2^)] (Table 5) signifying the bound probe to be more exposed and susceptible to solvent effects.

**TABLE 5:** SASA of ANS upon binding to formalin modified CHT (External Site).

Modification	SASA (Å^2^)	Binding site
Unmodified (Cys-1)	1,214	Near Cys-1
Cys-1	1,244.40	Toward Ser-119
Ser-119	1,236.36	Toward Ser-119
Asn-48	1,150/1,369	Over Cys-1 and Asn-48
Cys-1 Ser-119	1,402.37	Toward Ser-119
Cys-1 Asn-48	1,374.44	Over Asn-48
Ser-119 Asn-48	1,212.76/1,369	Toward Ser-119 & Over Asn-48
Cys-1 Ser-119 Asn-48	1,360.71	Over Asn-48

Similar to the binding interactions of substrate (AMC), residue modification at the internal ANS binding site in CHT (Ser-195) has no effect on binding of the probe (Figure 10). SASA analysis of ANS at the internal binding site of CHT suggests no major changes in solvent exposure of ANS (Table 6) suggesting that the bound probe is shielded from the surrounding solvent even after residue modification. [The calculated binding energies and cluster of poses are provided in the Supplementary Material]

**TABLE 6:** SASA of ANS upon binding to formalin modified CHT (Internal Site).

Modifications	SASA (Å^2^)
Unmodified Ser-195	1,027
Ser’	1,061
Ser’’	1,024
Ser’’’	1,013

### Residue modification disrupts activity and introduces heterogeneity in molecular recognition

CHT causes the catalytic hydrolysis of AMC to 7-amido 4-methyl-coumarin. (Biswas and Pal, 2004). The effect of formalin induced modification was monitored at different concentrations of formalin (1%–4%). We have observed a gradual decrease (Figure 2) in product formation with an increase in formalin concentration as well as an apparent fall in catalytic efficiency (Table 1). The Lineweaver-Burk plot (Supplementary Figure S1) and Table 1 indicates formalin exhibiting competitive inhibition (no change in Vmax and increasing K_m_) which may be inaccurate. Since formalin covalently modifies the active site residues causing irreversible covalent inhibition and can cause crosslinks (Marini and Martin, 1971; Martin et al., 1972; Tayri-Wilk et al., 2020) understanding the kinetic parameters of CHT using Lineweaver-Burk plot (Michealis-Menten kinetics) may not be accurate. A crucial assumption of the Michaelis–Menten model is that the total enzyme concentration does not change over time. As the active enzyme concentration changes due to formalin induced modification, the apparent k_cat_ and K_m_ values cannot be used to categorize the formalin mediated enzyme inhibition. Hence, whether formalin modification causes impairment of substrate binding at the active site cannot be discerned from Michaelis-Menten kinetics study of CHT under formalin modification. However, chymotrypsin goes through burst kinetics, we observed a longer initial phase for the formalin treated CHT (Supplementary Figure S2), suggesting retardation in the de-acylation of Ser-195 due to residue modification upon formalin treatment, which was also echoed in previous literature (Marini and Martin, 1971). Stability of the secondary and tertiary structure indicated to residue modification at the catalytic S1 pocket as the possible architect in reduction of catalytic activity of the enzyme in presence of formalin (Figure 3), further any change to the mass of the enzyme due to crosslinking would have reflected in its hydrodynamic diameter, which we found no evidence of in our DLS study. To support our hypothesis, we developed a molecular docking model to predict the cross-linking effects of active site residues on the binding of substrate. Previous studies by Martin et al. (1972), Zhang et al. (2014) found direct correlation between modification of the catalytic site residues and activity. Ser-195 at the catalytic triad was solvent exposed. Ser-195 modification and molecular docking **s**howed no change in substrate (AMC) binding at the catalytic triad (Figures 7,8). Furthermore, no major structural distortions in the binding pocket were observed (Supplementary Figure S2). The analysis implied a stable enzyme-substrate formation; however, disruption in the proton transfer cascade from Ser-195 to His-57 to Asp-102 due to serine-histidine cross-linking by formalin impairs product formation and release leading to the reduced catalytic activity. Molecular docking simulations further support that modifications of amino acid residues are solely responsible for the impaired catalysis.

Time resolved fluorescence studies of ANS in CHT manifest heterogeneity in binding of ANS upon formalin induced modification (Figures 4,5). As a result of formalin induced structural modification in the enzyme, ANS bound to an external binding site (Cys-1-122) (Figure 9) becomes increasingly exposed to water thereby leading to a decreased fluorescence lifetime and rotational relaxation time (Tables 3,4).

To understand the effects of amino acid residue modification on substrate and probe binding SASA calculations were performed. From our SASA calculations we identified four amino acid residues [Ser-195; Cys-1; Asn-48 & Ser-119] as possible centers residue modification for our study (Figure 6). Using the SASA analysis a molecular docking model was designed to understand the substrate and probe binding behavior upon formalin modification. Targeted molecular docking analysis of external probe binding site (Cys-1) indicated generation of new ANS binding sites having different solvent accessibility on formalin modification (Figure 9). However, no change in binding was observed for the internal binding site (Ser-195) of ANS in CHT. The SASA of bound ANS suggested the bound probe to be buried and shielded from the surrounding solvent (Tables 5,6). Our solvent effect and docking simulation studies of the bound probe was found to corroborate with the time resolved fluorescence anisotropy studies manifesting heterogeneity in ANS binding to CHT following formalin induced modification.

## Conclusion

The present work primarily focuses on the influence of residue modification on the catalytic activity and molecular recognition of CHT. The study found changes in the catalytic activity and molecular recognition of the enzyme (CHT) upon formalin induced chemical modification of its amino acid residues. The structural and molecular docking analysis provide evidence of no impediment to the formation of a stable enzyme-substrate complex due to formalin induced modifications; rather we assume the impairment of the proton transfer cascade in the catalytic triad due to Ser-195-His-57 cross-linking leads to reduced catalytic activity. Picosecond resolved fluorescence studies reveal generation of multiple binding sites of a fluorophore ANS in CHT, and upon treatment with formalin a reduction in fluorescence lifetime and rotational relaxation time of the probe is observed when it is bound to an external binding site. Subsequent molecular docking studies and SASA analysis provide evidence towards heterogeneity of ligand (ANS) interaction with the enzyme (CHT) upon amino acid residues. The present findings may in the future offer background regarding drug-target interaction, molecular recognition, macromolecular modification to generate new binding sites for enhanced ligand binding through protein engineering.

## Data Availability

The original contributions presented in the study are included in the article/Supplementary Material, further inquiries can be directed to the corresponding authors.
