# Supplementary material for: A combined spectroscopic and molecular modeling Study on structure-function-dynamics under chemical modification: Alpha-chymotrypsin with formalin preservative
Source: Front Chem. 2022 Aug 31;10:978668. doi: 10.3389/fchem.2022.978668 (PMC9473634; doi:10.3389/fchem.2022.978668)
Supplement: Supplementary file 1 [file DataSheet1.docx]

**Supplementary Information**

**A Combined Spectroscopic and Molecular Modeling Study on Structure-Function-Dynamics under Chemical Modification: Alpha-Chymotrypsin with Formalin Preservative**

Pritam Biswas^1^, Aniruddha Adhikari^2^, Uttam Pal^3^, Susmita Mondal^2^, Dipanjan Mukherjee^2^, Ria Ghosh^3^, Rami J. Obaid^4^, Ziad Moussa^5^, Sudeshna Shyam Choudhury^1^*, Saleh A. Ahmed^4,6*^, Ranjan Das^7^*, Samir Kumar Pal^2,3^*

*^1^Department of Microbiology,*

*St. Xavier’s College,*

*30, Mother Teresa Sarani,*

*Kolkata 700016, India*

*^2^Department of Chemical, Biological and Macromolecular Sciences,*

*S. N. Bose National Centre for Basic Sciences,*

*Block JD, Sector III, Salt Lake,*

*Kolkata 700106, India*

*^3^Technical Research Centre,*

*S. N. Bose National Centre for Basic Sciences,*

*Block JD, Sector III, Salt Lake,*

*Kolkata 700106, India*

*^4^Department of Chemistry, Faculty of Applied Sciences*

*Umm Al-Qura University, Makkah 21955, Saudi Arabia*

*^5^Department of Chemistry, College of Science*

*United Arab Emirates University, P.O. Box 15551,*

*Al Ain, Abu Dhabi, United Arab Emirates*

*^6^Department of Chemistry, Faculty of Science*

*Assiut University, Assiut 71516, Egypt*

*^7^Department of Chemistry,*

*West Bengal state University,*

*Barasat, Kolkata 700126, India*

Corresponding Author E-mail: [skpal@bose.res.in](mailto:skpal@bose.res.in) (S. K. Pal)

[ranjan.das68@gmail.com](mailto:ranjan.das68@gmail.com) (R. Das)

[saahmed@uqu.edu.sa](mailto:saahmed@uqu.edu.sa) (S. A. Ahmed)

[sudeshna.s.choudhury@sxccal.edu](mailto:sudeshna.s.choudhury@sxccal.edu)(S. S. Choudhury)

**Supplementary Figure**

**
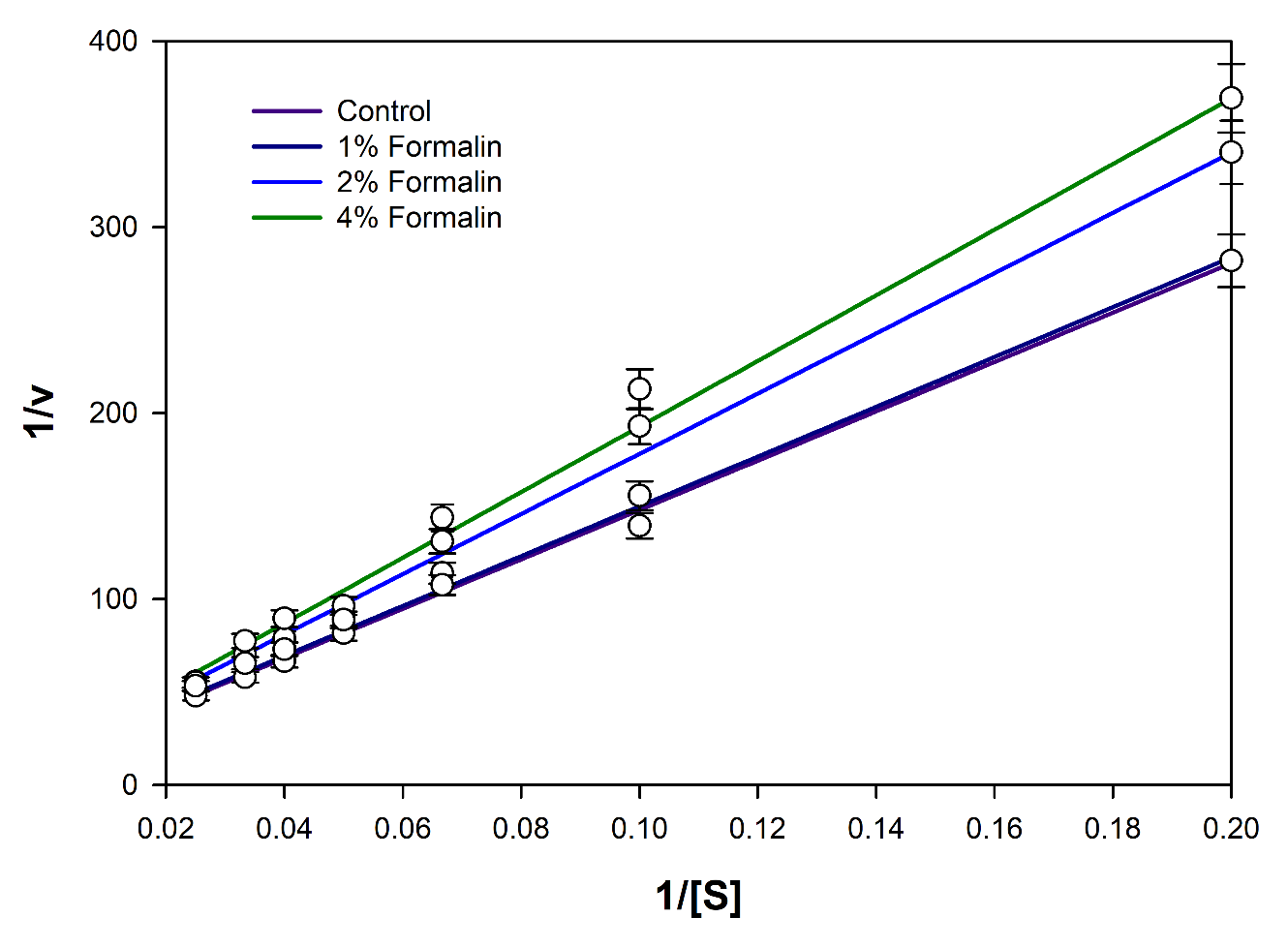
**

**Supplementary Figure. S1: Showing the Lineweaver-Burk plot of the enzyme CHT at different formalin concentration upon substrate (AMC) hydrolysis.**


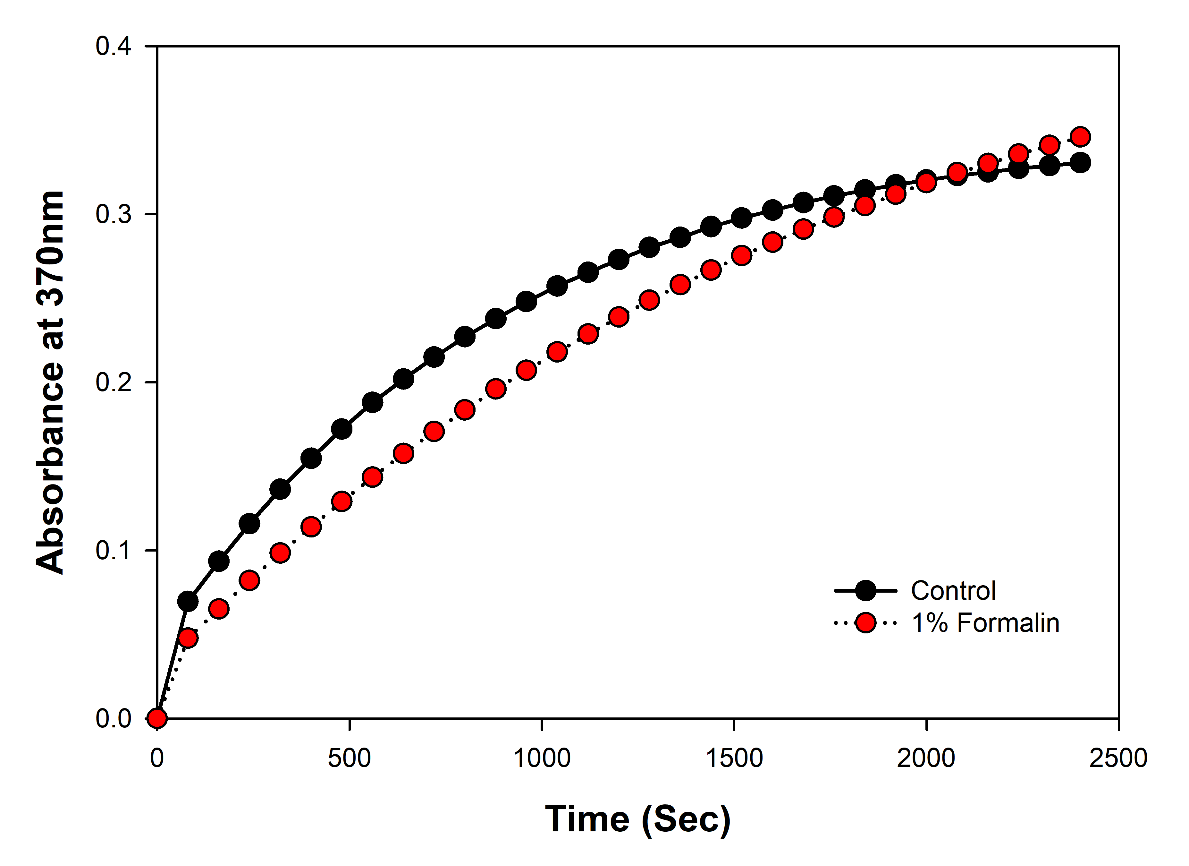


**Supplementary Figure S2: Representative figure showing the absorbance at 370nm of the product (7-amido 4-methylcoumarin) for control and 1% formalin.**


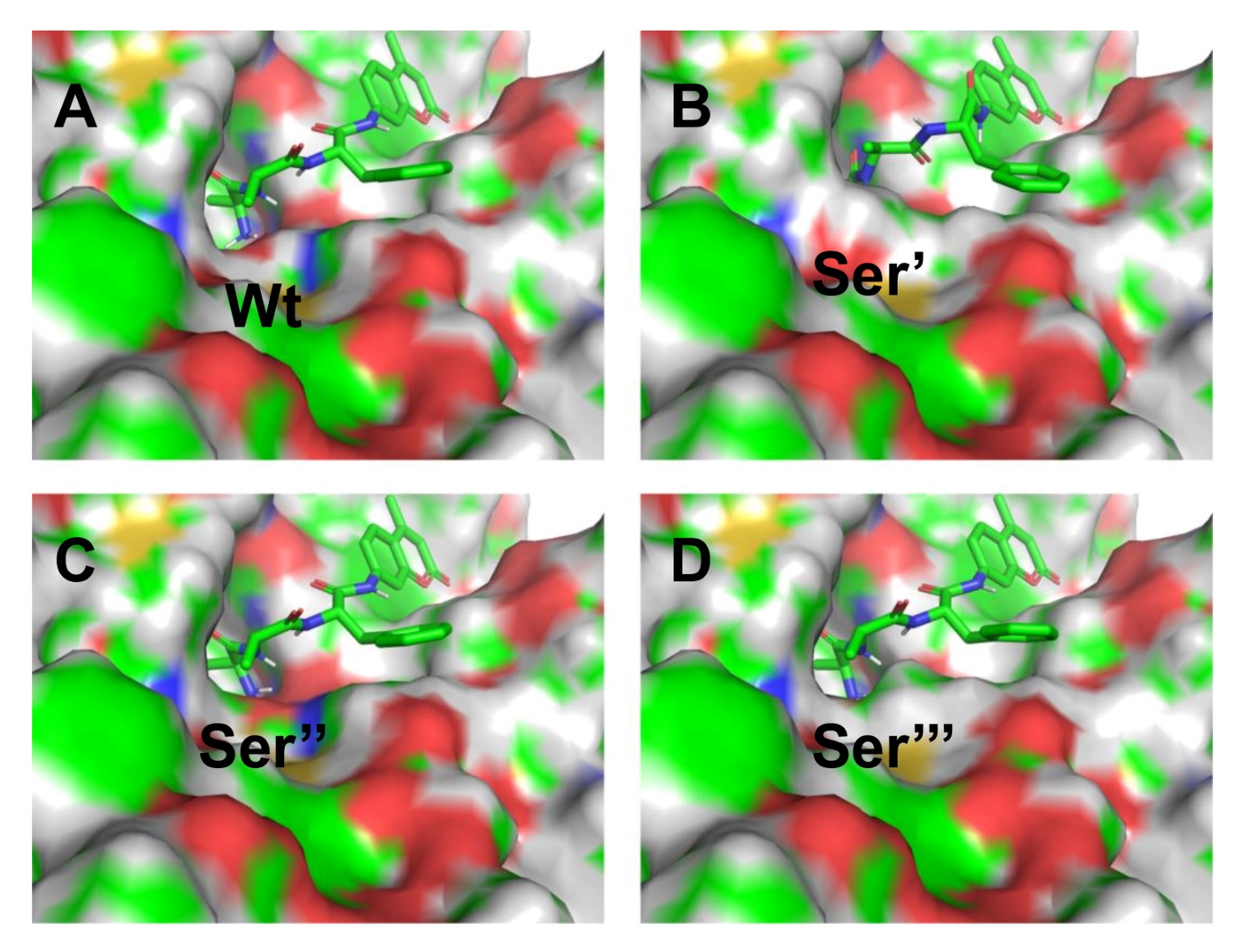


**Supplementary Figure S3: Surface view of the binding cavity at Wt and HCHO modified CHT. (A-D) Insignificant changes to the binding cavity upon residue modification at Ser-195.**


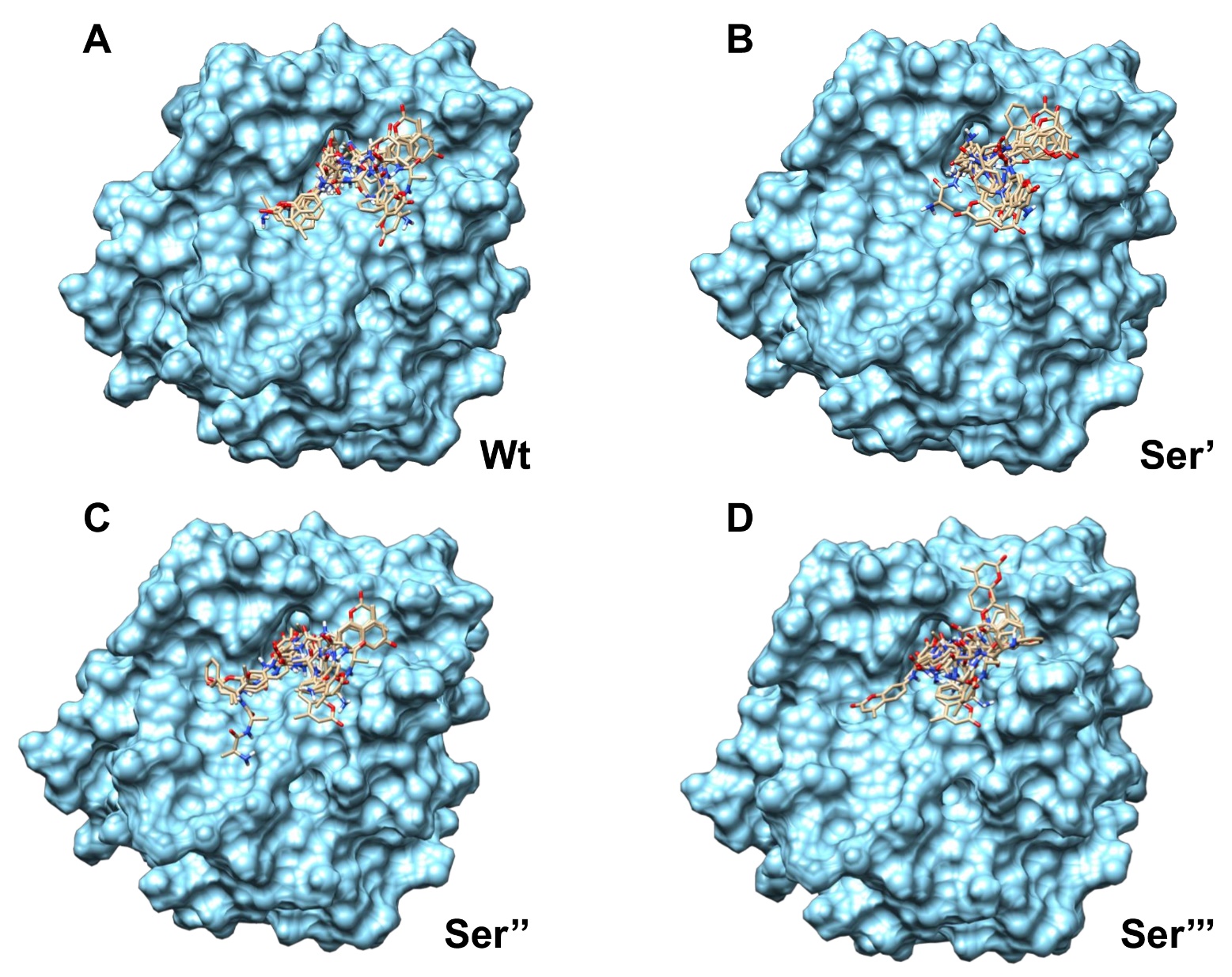


**Supplementary Figure S4: Showing the different binding pose between substrate AMC and formalin modified CHT (A-D) at the substrate binding site.**


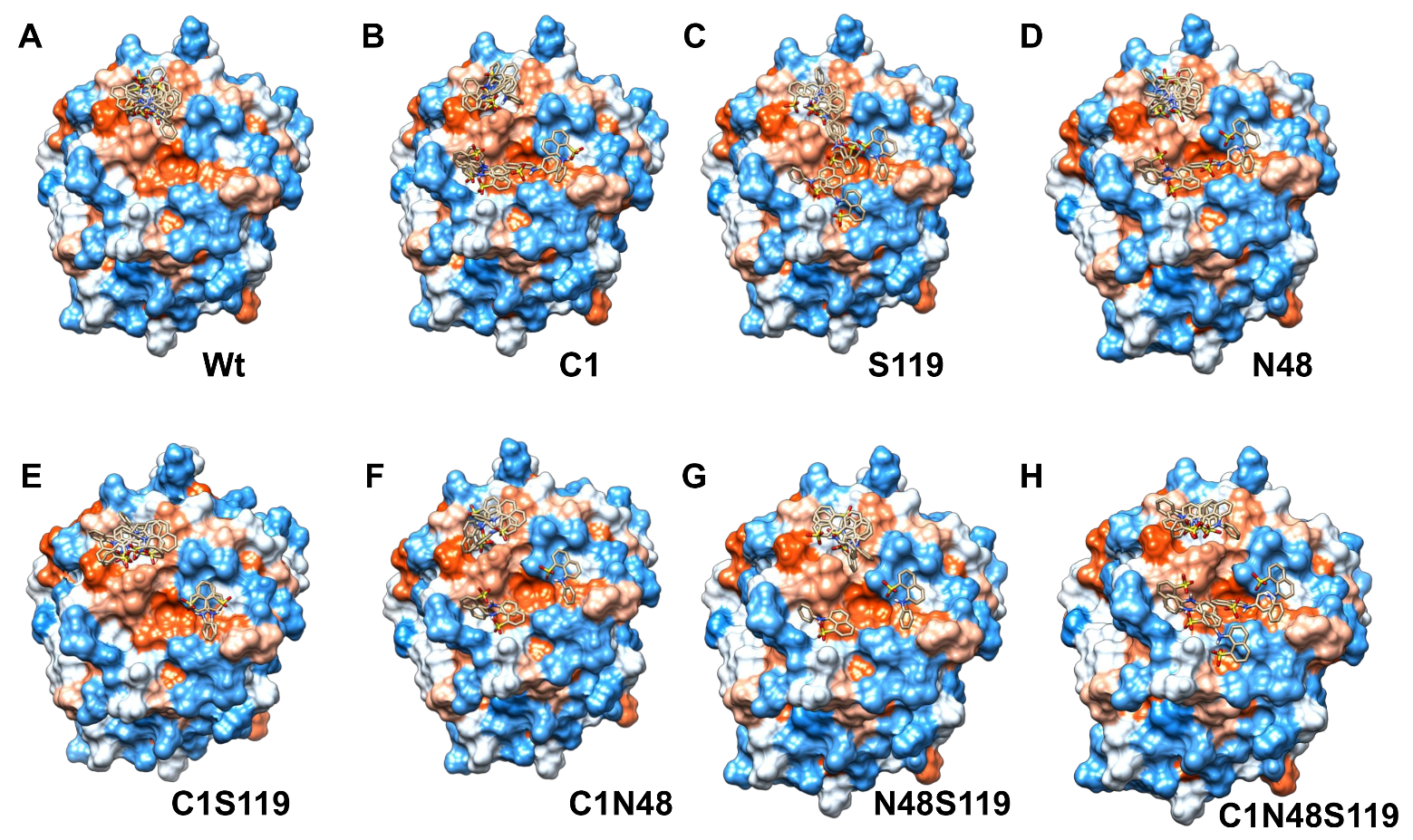


**Supplementary Figure S5: Showing the binding pose of ANS between the probe ANS and formalin modified CHT (A-H) at the probe binding site (External).**


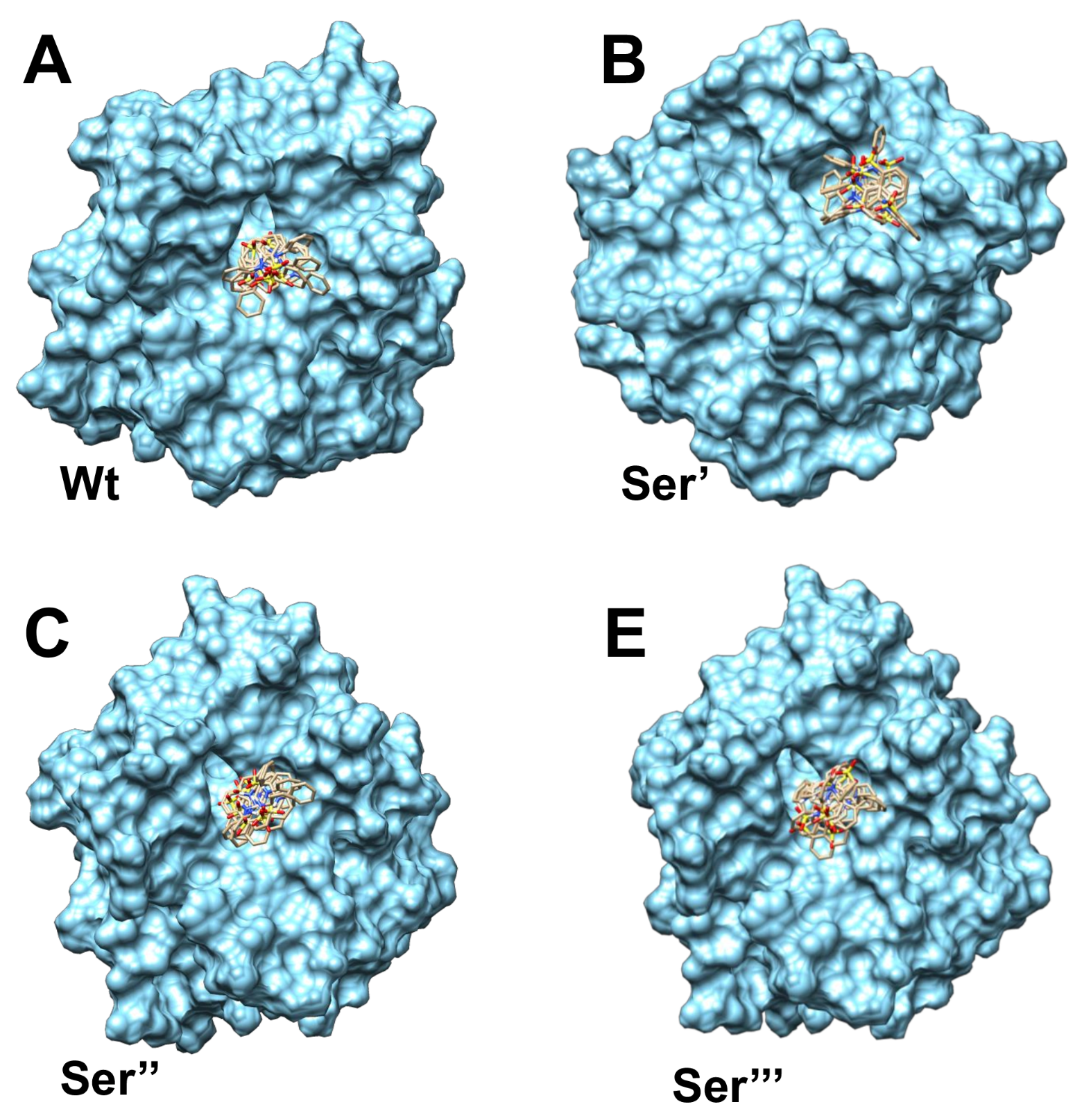


**Supplementary Figure S6: Showing the binding pose of ANS between the probe ANS and formalin modified CHT (A-D) at the probe binding site (Internal).**

**Supplementary Table**

**Table.S1: Globular Tertiary Structure of alpha-Chymotrypsin upon Formalin Treatment**

| Formalin conc. (%) | Hydrodynamic Diameter (nm) |
| --- | --- |
| Control | ~ 7.0 |
| 1 | ~6.0 |
| 2 | ~ 6.0 |
| 4 | ~ 5.0 |

**Table S2a: Binding energies of different binding poses for AMC-CHT**

| Wt | Binding Energy(kcal/mol) |
| --- | --- |
|  | -8.3 |
|  | -8.2 |
|  | -8.0 |
|  | -8.0 |
|  | -7.9 |
|  | -7.8 |
|  | -7.8 |
|  | -7.8 |
|  | -7.7 |

**Table S2b: Binding energies of different binding poses for AMC-CHT**

| Ser’ | Binding Energy(kcal/mol) |
| --- | --- |
|  | -8.4 |
|  | -8.4 |
|  | -8.4 |
|  | -8.3 |
|  | -8.1 |
|  | -8.0 |
|  | -8.0 |
|  | -7.8 |
|  | -7.7 |

**Table S2c: Binding energies of different binding poses for AMC-CHT**

| Ser’’ | Binding Energy(kcal/mol) |
| --- | --- |
|  | -8.4 |
|  | -8.0 |
|  | -8.0 |
|  | -7.9 |
|  | -7.9 |
|  | -7.9 |
|  | -7.9 |
|  | -7.6 |
|  | -7.6 |

**Table S2d: Binding energies of different binding poses between AMC-CHT**

| Ser’’’ | Binding Energy(kcal/mol) |
| --- | --- |
|  | -8.3 |
|  | -7.9 |
|  | -7.9 |
|  | -7.9 |
|  | -7.8 |
|  | -7.7 |
|  | -7.3 |
|  | -7.1 |
|  | -7.0 |

**Table S3a: Binding energies of different binding poses between ANS-CHT (External)**

| Wt | Binding Energy(kcal/mol) |
| --- | --- |
|  | -5.4 |
|  | -5.2 |
|  | -5.2 |
|  | -5.1 |
|  | -5.1 |
|  | -5.0 |
|  | -5.0 |
|  | -4.8 |
|  | -4.8 |

**Table S3b: Binding energies of different binding poses between ANS-CHT (External)**

| C1 | Binding Energy(kcal/mol) |
| --- | --- |
|  | -5.0 |
|  | -4.9 |
|  | -4.9 |
|  | -4.8 |
|  | -4.7 |
|  | -4.6 |
|  | -4.6 |
|  | -4.6 |
|  | -4.5 |

**Table S3c: Binding energies of different binding poses between ANS-CHT (External)**

| S119 | Binding Energy(kcal/mol) |
| --- | --- |
|  | -5.2 |
|  | -5.2 |
|  | -4.9 |
|  | -4.9 |
|  | -4.8 |
|  | -4.7 |
|  | -4.6 |
|  | -4.6 |
|  | -4.6 |

**Table S3d: Binding energies of different binding poses between ANS-CHT (External)**

| N48 | Binding Energy(kcal/mol) |
| --- | --- |
|  | -5.3 |
|  | -5.3 |
|  | -5.2 |
|  | -4.8 |
|  | -4.8 |
|  | -4.8 |
|  | -4.8 |
|  | -4.7 |
|  | -4.7 |

**Table S3e: Binding energies of different binding poses between ANS-CHT (External)**

| N48S119 | Binding Energy(kcal/mol) |
| --- | --- |
|  | -5.2 |
|  | -5.2 |
|  | -5.1 |
|  | -4.9 |
|  | -4.8 |
|  | -4.8 |
|  | -4.7 |
|  | -4.7 |
|  | -4.6 |

**Table S3f: Binding energies of different binding poses between ANS-CHT (External)**

| C1S119 | Binding Energy(kcal/mol) |
| --- | --- |
|  | -5.1 |
|  | -5.0 |
|  | -4.9 |
|  | -4.8 |
|  | -4.8 |
|  | -4.8 |
|  | -4.8 |
|  | -4.7 |
|  | -4.7 |

**Table S3g: Binding energies of different binding poses between ANS-CHT (External)**

| C1N48 | Binding Energy(kcal/mol) |
| --- | --- |
|  | -5.2 |
|  | -5.0 |
|  | -4.9 |
|  | -4.9 |
|  | -4.7 |
|  | -4.7 |
|  | -4.7 |
|  | -4.6 |
|  | -4.5 |

**Table S3h: Binding energies of different binding poses between ANS-CHT (External)**

| C1N48S119 | Binding Energy(kcal/mol) |
| --- | --- |
|  | -5.3 |
|  | -5.0 |
|  | -4.9 |
|  | -4.8 |
|  | -4.7 |
|  | -4.7 |
|  | -4.7 |
|  | -4.7 |
|  | -4.6 |

**Table S4a: Binding energies of different binding poses between ANS-CHT (Internal)**

| Wt | Binding Energy(kcal/mol) |
| --- | --- |
|  | -6.4 |
|  | -6.2 |
|  | -5.8 |
|  | -5.8 |
|  | -5.7 |
|  | -5.7 |
|  | -5.2 |
|  | -5.2 |
|  | -5.2 |

**Table S4b: Binding energies of different binding poses between ANS-CHT (Internal)**

| Ser’ | Binding Energy(kcal/mol) |
| --- | --- |
|  | -6.5 |
|  | -6.3 |
|  | -6.0 |
|  | -5.8 |
|  | -5.8 |
|  | -5.7 |
|  | -5.7 |
|  | -5.6 |
|  | -5.2 |

**Table S4c: Binding energies of different binding poses between ANS-CHT (Internal)**

| Ser’’ | Binding Energy(kcal/mol) |
| --- | --- |
|  | -6.4 |
|  | -6.2 |
|  | -6.0 |
|  | -5.8 |
|  | -5.7 |
|  | -5.7 |
|  | -5.4 |
|  | -5.2 |
|  | -5.2 |

**Table S4d: Binding energies of different binding poses between ANS-CHT (Internal)**

| Ser’’’ | Binding Energy(kcal/mol) |
| --- | --- |
|  | -6.5 |
|  | -6.2 |
|  | -6.0 |
|  | -5.8 |
|  | -5.7 |
|  | -5.7 |
|  | -5.6 |
|  | -5.6 |
|  | -5.4 |
